# Reproducibility of chestwall and heart position using surface‐guided versus RPM‐guided DIBH radiotherapy for left breast cancer

**DOI:** 10.1002/acm2.13755

**Published:** 2022-08-22

**Authors:** Wei Lu, Guang Li, Linda Hong, Ellen Yorke, Xiaoli Tang, James G. Mechalakos, Pengpeng Zhang, Laura I. Cerviño, Simon Powell, Sean L. Berry

**Affiliations:** ^1^ Department of Medical Physics Memorial Sloan Kettering Cancer Center New York New York USA; ^2^ Department of Radiation Oncology Memorial Sloan Kettering Cancer Center New York New York USA

**Keywords:** breast cancer, comparison, deep inspiration breath hold, real‐time position management, surface imaging

## Abstract

This study compared the reproducibility of chestwall and heart position using surface‐guided versus RPM (real‐time position management)‐guided deep inspiration breath hold (DIBH) radiotherapy for left sided breast cancer. Forty DIBH patients under either surface‐guided radiotherapy (SGRT) or RPM guidance were studied. For patients treated with tangential fields, reproducibility was measured as the displacements in central lung distance (CLD) and heart shadow to field edge distance (HFD) between pretreatment MV (megavoltage) images and planning DRRs (digitally reconstructed radiographs). For patients treated with volumetric modulated arc therapy (VMAT), sternum to isocenter (ISO) distance (StID), spine to rib edge distance (SpRD), and heart shadow to central axis (CAX) distance (HCD) between pretreatment kV images and planning DRRs were measured. These displacements were compared between SGRT and RPM‐guided DIBH. In tangential patients, the mean absolute displacements of SGRT versus RPM guidance were 0.19 versus 0.23 cm in CLD, and 0.33 versus 0.62 cm in HFD. With respect to planning DRR, heart appeared closer to the field edge by 0.04 cm with surface imaging versus 0.62 cm with RPM. In VMAT patients, the displacements of surface imaging versus RPM guidance were 0.21 versus 0.15 cm in StID, 0.24 versus 0.19 cm in SpRD, and 0.72 versus 0.41 cm in HCD. Heart appeared 0.41 cm further away from CAX with surface imaging, whereas 0.10 cm closer to field CAX with RPM. None of the differences between surface imaging and RPM guidance was statistically significant. In conclusion, the displacements of chestwall were small and were comparable with SGRT‐ or RPM‐guided DIBH. The position deviations of heart were larger than those of chestwall with SGRT or RPM. Although none of the differences between SGRT and RPM guidance were statistically significant, there was a trend that the position deviations of heart were smaller and more favorable with SGRT than with RPM guidance in tangential patients.

## 1 INTRODUCTION

Radiation therapy (RT) is an important treatment modality for many patients with breast cancer. For left breast patients, RT often spreads some incidental radiation dose to the heart. It was shown that the rates of major coronary events increased linearly with the mean dose to the heart by 7.4% per gray, with no apparent threshold.^1^ To reduce the heart dose, one effective approach is to treat patients with deep inspiration breath‐hold (DIBH) as it often increases the distance between the chest wall and heart.^2^, ^3^, ^4^ A review of 18 studies summarized that DIBH reduced the mean heart dose by 26.2% to 75.0%.^3^ A review of 41 studies with a total of 3599 patients showed that DIBH reduced heart dose, left anterior descending branch (LAD) dose, ipsilateral lung dose, and heart volume significantly.^5^ A prospective study indicated that none of the 20 DIBH patients had post‐RT cardiac perfusion or wall motion abnormalities at 6 months.^6^ Furthermore, recent studies showed that DIBH reduced doses to almost all cardiac substructures, particularly to LAD and left ventricle, which could potentially translate into the clinical benefit of reduced cardiac toxicity.^7^, ^8^ The reduction of cardiac doses was similar in patients with modified radical mastectomy as well as with breast conservation surgery.^9^ Secondary advantages of DIBH include reducing relative volume of lung exposed to radiation and minimizing respiratory motion.^2^, ^10^

The earliest DIBH was implemented with spirometry‐based systems including voluntary DIBH^11^, ^12^ and active breathing control.^13^, ^14^, ^15^ A more widely used technique is voluntary DIBH guided with the video‐based real‐time position management (RPM) system (Varian Medical Systems, Palo Alto, CA).^3^, ^16^, ^17^, ^18^, ^19^ Recently, 3D optical surface imaging systems such as AlignRT (VisionRT, London, UK) are also used to guide voluntary DIBH as surface‐guide radiotherapy (SGRT).^4^, ^6^, ^20^, ^21^, ^22^, ^23^, ^24^, ^25^, ^26^, ^27^, ^28^ With these techniques, the treatment beam is held if the breath hold is outside preset tolerance levels. Being a 3D system, SRGT is expected to have potential to improve the setup accuracy and the reproducibility of the DIBH position.^20^, ^24^, ^29^ It became an important question how the SGRT system actually performs compared to the RPM system to support more adoption of this new technique. We found few studies in the literature comparing the two systems; these include studies in phantom^30^, ^31^ and 10 thoracic patients^32^, ^33^ for 4DCT and a study in four tangential breast patients for DIBH.^21^ In this study, we compared displacement of chestwall and heart with SGRT‐guided versus RPM‐guided DIBH in 40 patients with left breast cancer. Both tangential and volumetric modulated arc therapy (VMAT) patients were studied.

## 2 METHODS AND MATERIALS

### 2.1 Patients

This retrospective study is approved by our institutional review board. A total of 40 left breast or left chestwall patients were evaluated. Twenty patients with early‐stage cancer were treated for whole breast irradiation with tangential fields, and the other 20 with nodal involvement were treated with VMAT from 2015 to 2020. These patients were treated at two separate sites in our institution, 20 at site 1 equipped with the RPM system and 20 at site 2 with a SGRT system (AlignRT, Vision RT Ltd., London, UK). The two sites follow the same radiotherapy procedures (to the maximal extent as possible), institution‐wide therapist, and physics competencies are administered, all patients are evaluated at the same chart rounds, and we cross‐train staff at different sites; therefore we expect the same quality and competence between sites.

### 2.2 Patient setup and breath hold monitoring

All patients were positioned with a CIVCO breast board (CIVCO Medical Solutions, Coralville, Iowa) with both arms above the head and a foam roll under the knees. Patients were scanned with 16‐slice Philips Brilliance CT scanners (Philips Healthcare, Amsterdam, Netherlands) for treatment simulation. At site 1 the Varian RPM system was used to monitor patient breathing during CT simulation and at treatment. Only audio guidance for breathing hold was provided to the patient. RPM reflective block was positioned along the body midline close to the xiphoid process, and its position was indicated in the setup instructions with respect to castlines or the tattooed isocenter. Prior to the simulation scan, patients were coached to follow a modified slow vital capacity maneuver – normal tidal breathing for a few cycles, then a deep inhale followed by a deep exhale followed by a deep inhale, which is held at a level close to peak inspiration (DIBH) for 15–20 s. This was monitored with the RPM system and repeated for a few times to familiarize patients with the process. For the planning CT scan, patients were coached into this maneuver, and the CT was acquired during the breath‐hold. Upper and lower gating threshold lines were set to 0.25 cm above and below the average level of the breath hold. This defined a gating window of 0.5 or ±0.25 cm. This breathing trace with the gate was transferred to the treatment machine to assure the patient would hold the breath in the same gating window as at CT simulation for daily treatment. Another free breathing (FB) CT scan was performed immediately following the DIBH scan, and patients were instructed to not move between the two scans. The FB scan was used for the setup to skin tattoos and for backup, in case the patient was unable to perform DIBH at some point in treatment.

At site 2 the bellows system (Philips Healthcare, Amsterdam, Netherlands) was used to monitor patient breathing at CT simulation, and the SGRT system (AlignRT) was used at treatment. The same coaching technique described above with the RPM system was used with audio guidance. A treatment plan was constructed (see II.C) and was exported along with the body contour to AlignRT. In AlignRT a large region of interest (ROI)^34^ was drawn on the body surface to serve as the setup reference image. The ROI included the entire ipsilateral breast with some extensions as illustrated in Li et al.^34^ and Alderliesten et al.^20^ Specifically, the ROI was defined with four boundaries: from the body midline in the sagittal view on the ipsilateral side to the nipple of the contralateral breast in the medial–lateral direction and from the supraclavicular match‐line to 2 cm below the breast tissue in the superior–inferior direction.^34^ Gating thresholds were ±0.3 cm translation and ±3.0° rotation in each of the three directions, and thus is 6D. The same ROI definition and gating thresholds were used for both tangential and VMAT patients.

### 2.3 Contouring and treatment planning

The MIM (MIM Software Inc. Cleveland, OH) and the Eclipse (Varian Medical Systems, Palo Alto, CA) systems were used for contouring and treatment planning, respectively. Both contouring and planning were done on the DIBH CT scan. Patients with early‐stage cancer (breast only) were treated with two tangential beams (medial and lateral), while patients with nodal involvement (breast + regional nodes) were treated with VMAT. For tangential plans, physicians contoured the heart, and planners contoured the left lung, nipple, and left breast. The prescription was 4240 cGy in 16 fractions. A tangential plan was constructed by using a 3D conformal technique with an in‐house dynamic compensator design software.^35^ The resulting optimal fluence was imported into Eclipse where leaf motion and dose were calculated. For VMAT plans, additional structures were contoured, including CTVs and PTVs by physicians, right lung, and esophagus by planners. The prescription was 5000 cGy in 25 fractions or 4256 cGy in 16 fractions. Both breast and regional nodes (internal mammary, supraclavicular, and axillary lymph nodes) were treated with four or five partial arcs (with 60–100 degrees span), which were planned and optimized with Eclipse VMAT optimization.^36^ A bolus (0.3 cm thick) covering the entire RT field was applied in Eclipse for every VMAT patient to assure adequate skin dose. For RPM, conventional SuperFlab bolus was used. For AlignRT, Elasto‐Gel bolus (Radiation Products Design, Inc Albertville, MN) with one side built‐in white fabric was used to visualize the light projected by the SGRT system.

### 2.4 Online imaging and data analysis

Patients were treated with Varian linacs including 600EX, Trilogy, and True Beam models (Varian Medical Systems, Palo Alto, CA). Figure 1 in ref. [34] illustrates the complete workflow for AlignRT treatment at our institution. With either RPM‐ or AlignRT‐guidance, patients were first set up free‐breathing based on skin tattoos. With RPM guidance, the patient was coached to perform a DIBH, and therapists checked that the RPM breathing trace fell into the gating window. With AlignRT‐guidance, the patient was set up with a two‐step procedure every day^34^: (1) align the arm and chin, (2) align the FB CT reference surface ROI by applying couch shifts in 6D. After the FB setup, the patient was coached to perform a DIBH while the couch lateral and longitudinal positions were adjusted to match the DIBH CT reference surface ROI, that is, to make the real‐time delta fall into the gating window of ±0.3 cm translation and ±3.0° rotation. Couch's vertical position was kept unchanged. Occasionally patients’ DIBH did not fall into the gating window with either RPM‐ or AlignRT‐guidance, this was usually resolved after therapists talked to the patients and coached patients for another DIBH. Once the DIBH setup was satisfactory, a new “live surface” image was always acquired (regardless of bolus application) and was used for that day only to monitor DIBH motion. If bolus was prescribed, therapists coached the patient to perform a DIBH, placed the bolus quickly, and then immediately acquired a “live surface” as new reference, all within the same DIBH.

**FIGURE 1:**
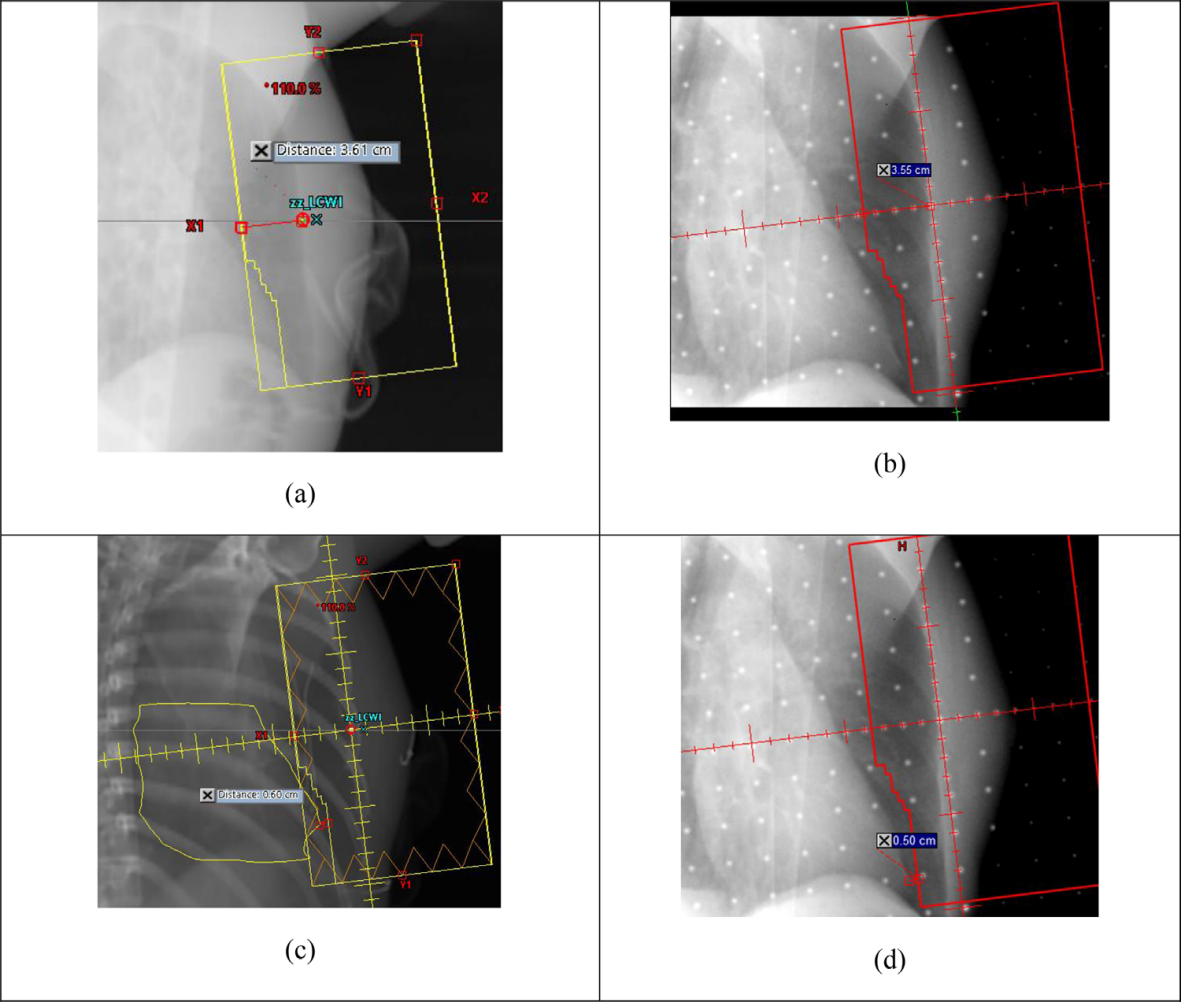
Example tangential patient. Central lung distance (CLD) and heart shadow to field edge distance (HFD) measured in DRRs (a), (c), and MV portal images (b), (d), respectively. Yellow contour in (c) is heart (including pericardial fat if presented). ΔCLD = 3.55–3.61 = ‐0.06 cm and ΔHFD = 0.50–0.60 = −0.10 cm

Therapists then proceeded to online imaging. For tangential patients, weekly MV portal images were acquired with chestwall alignment to DRR as setup criteria. Both medial and lateral portal images were acquired on the first day, while only medial portal images were acquired in the following weeks. For VMAT patients, daily kV orthogonal images (anterior/posterior [AP] and lateral [LAT]) were acquired with anterior sternum and chestwall ribcage alignment to DRR as setup criteria. For RPM‐guided DIBH, image acquisitions were automatically triggered after a 0.5 s delay from when a patient reached the lower gating threshold line. With the 0.5 s delay, images were acquired at a breath‐hold level closer to the average or middle level between the upper and lower gating lines. For AlignRT‐guided DIBH, image acquisitions were manually triggered by therapists when they saw that patient's breathing fell into the gating window. The final couch shifts were based on MV or kV image alignment. Heart position in the MV or kV images was observed but not adjusted or used in determining the couch shifts. When consistent and large heart displacements were observed, the physician was notified, and the fields could be adjusted in a revised plan.

**FIGURE 2:**
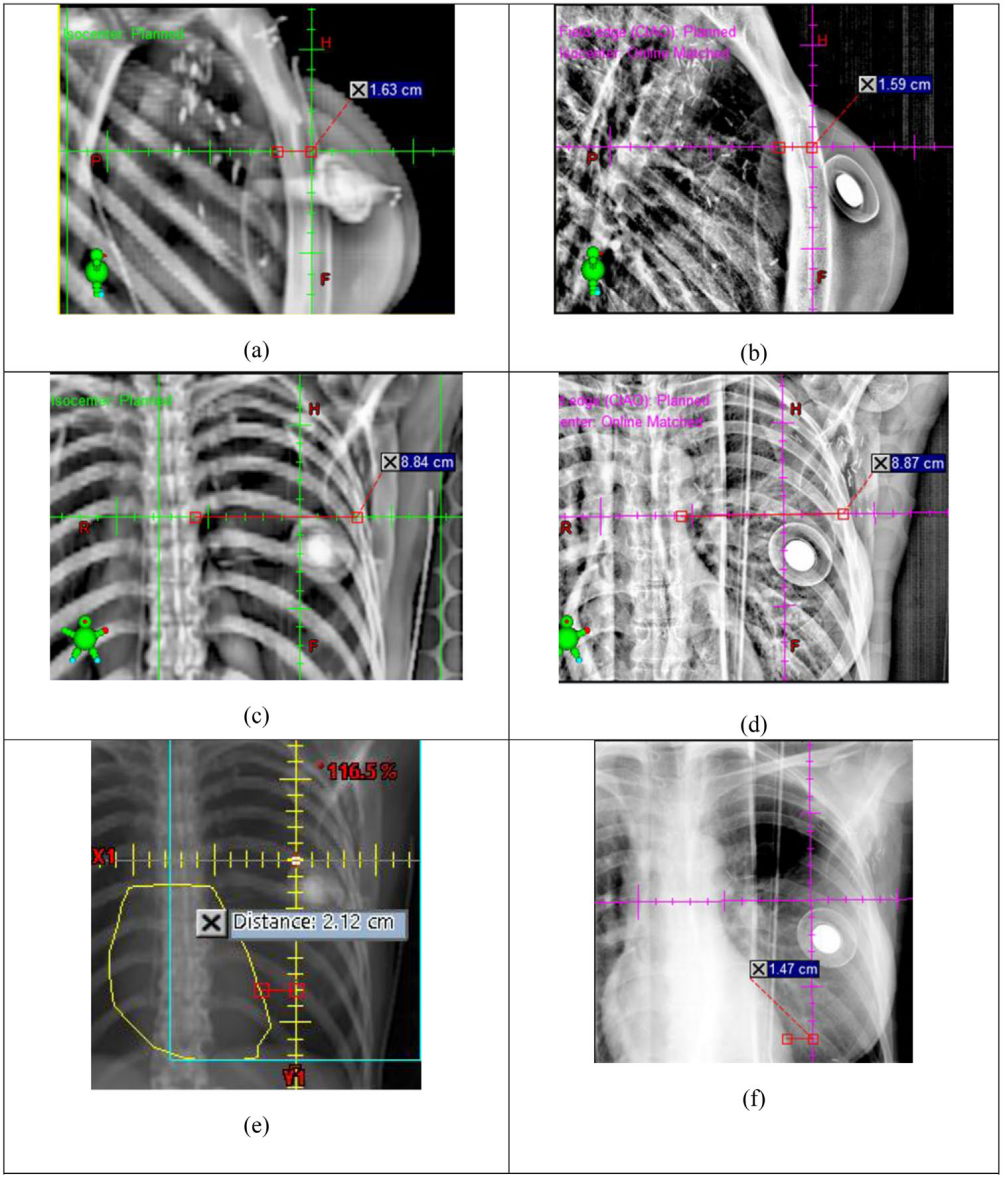
Example volumetric modulated arc therapy (VMAT) patient. Sternum to ISO distance (StID), Spine to Rib Edge Distance (SpRD), and Heart Shadow to Central Axis Distance (HCD) measured in DRRs (a), (c), (e), and AP/LAT kV setup images (b), (d), (f), respectively. ΔStID = 1.59–1.63 = −0.04 cm, ΔSpRD = 8.87–8.84 = 0.03 cm, and ΔHCD = 1.47–2.12 = −0.65 cm

We made the following measurements on weekly basis: on MV images for tangential patients (Figure 1): central lung distance (CLD)—the distance from chestwall to posterior field edge. CLD measures the chestwall position and the amount of lungs in the field. Heart shadow to field edge distance (HFD)—the distance from the most anterior heart shadow to posterior field edge. HFD measures how far the heart is away from the field (positive HFD) or inside the field (negative HFD). On kV images for VMAT patients (Figure 2): sternum to ISO distance (StID)—the distance from the posterior base of the sternum to field ISO. Spine to Rib Edge Distance (SpRD)—the distance from the lateral spine to the inner rib edge. SpRD measures lung expansion. Both StID and SpRD were measured along the central axis (CAX). Heart shadow to CAX distance (HCD), measured at a specific superior/inferior (SI) vertebral position (range: top of T8 to top of T11) for each patient. The heart spans T5–T8 at rest and T7–T11 at DIBH. If pericardial fat was present, it was contoured and combined with the heart contour for locating the “heart” shadow. The image display filter was chosen to optimally measure each of the distances and was kept the same for all patients. The differences (Δ) of these distances between the MV/kV images and corresponding DRRs were calculated to evaluate the displacement of chestwall and heart from planning to treatment. To compare the displacement between RPM‐guided DIBH and AlignRT‐guided DIBH, the analysis of variance (ANOVA) test for repeated measurements was run in SAS software (SAS Institute). This test also assesses if there are any differences among the repeated measurements for each technique. The difference was considered statistically significant if the *p*‐value <0.05. A correlation coefficient *r* > 0.7 was considered high, whereas *r* < 0.3 was considered low.

## 3 RESULTS

### 3.1 Displacement of chestwall and heart in tangential patients

**FIGURE 3:**
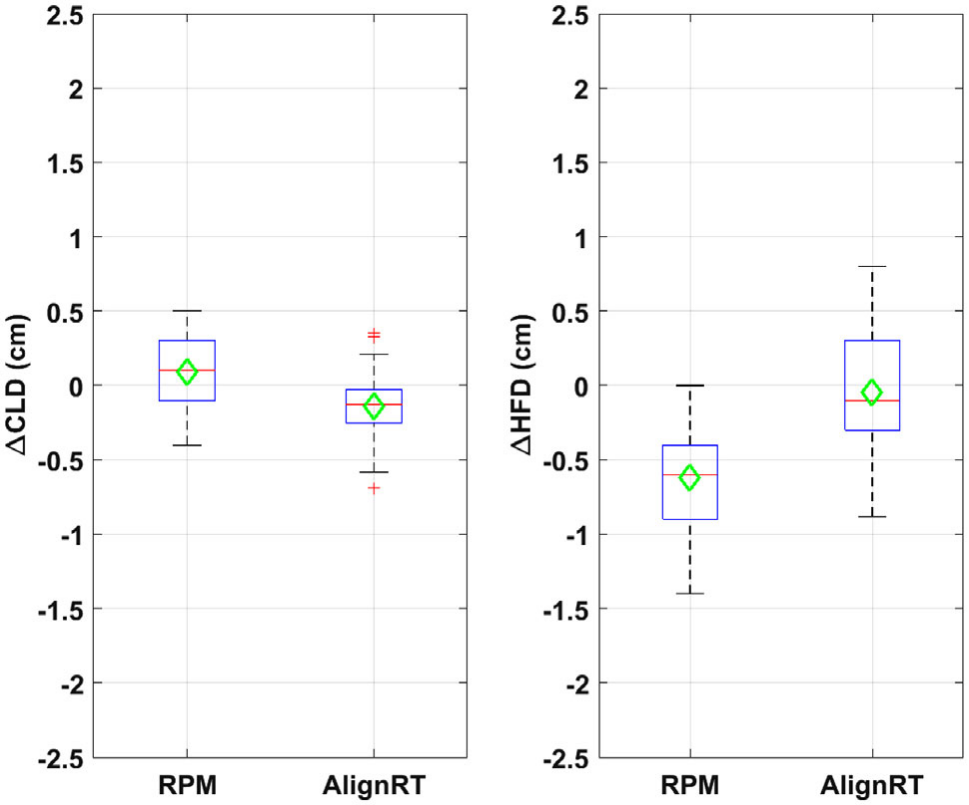
Box plots of ΔCLD and ΔHFD with RPM‐ and AlignRT‐guided deep inspiration breath hold (DIBH). The central line/marker indicates the median/mean, and the box indicates the 25th and 75th percentiles

**FIGURE 4:**
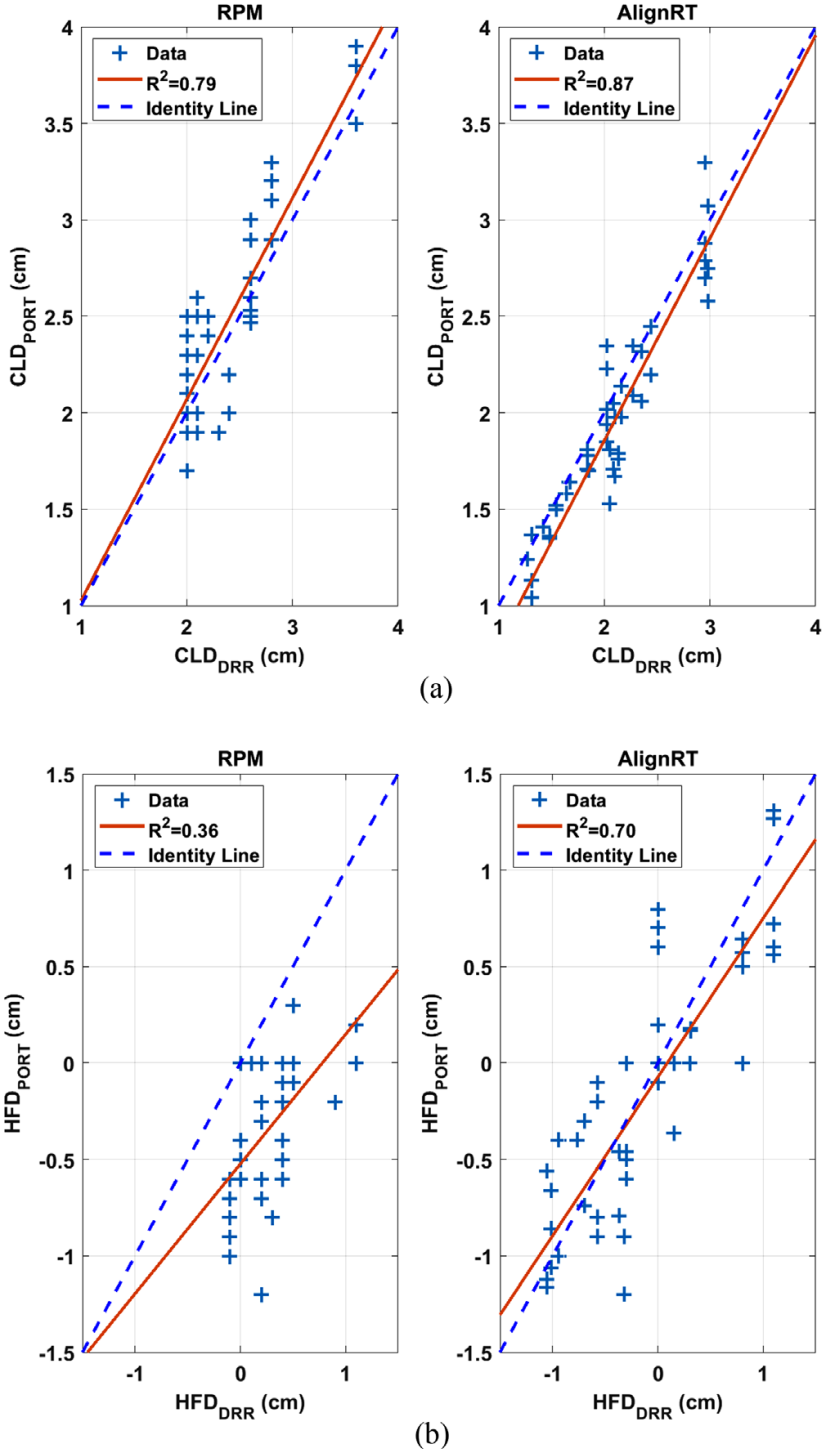
Correlation between (a) CLD_DRR_ and CLD_PORT_ and between (b) HFD_DRR_ and HFD_PORT_ in tangential patients. The dashed line is the identity line.

Table 1 and Figure 3 show the displacement in CLD and HFD in tangential patients. MV portal images were acquired once a week for 3–4 weeks. At least four images for each patient, and a total of 42 images for RPM patients and 46 images for AlignRT patients were analyzed. The mean (absolute) displacement was the (absolute) displacement averaged across all patients and fractions. The mean ΔCLDs were small for both RPM and AlignRT systems (0.09 and −0.14 cm), suggesting there was no systematic displacement of chestwall. The mean absolute displacement in CLD was small and comparable with either RPM (0.23 cm) or AlignRT (0.19 cm). The correlation between CLD_DRR_ and CLD_PORT_ (Figure 4) was slightly lower with RPM guidance (*R* ^2^ = 0.79, *r* = 0.89) than with AlignRT guidance (*R* ^2^ = 0.87, *r* = 0.93).

**TABLE 1:** Displacement in tangential patients

Displacement (cm)		RPM (*N* = 42)	AlignRT (*N* = 46)	*p*‐Value ANOVA
ΔCLD	Mean ± SD	0.09 ± 0.26	−0.14 ± 0.21	0.35
	Median (IQR)	0.10 (−0.10, 0.30)	−0.13 (−0.25, −0.03)	
	Abs. mean ± SD	0.23 ± 0.15	0.19 ± 0.16	0.63
	Abs. median (IQR)	0.20 (0.10, 0.30)	0.16 (0.05, 0.29)	
ΔHFD	Mean ± SD	−0.62 ± 0.31	−0.04 ± 0.39	0.75
	Median (IQR)	−0.60 (‐0.90, ‐0.40)	−0.10 (−0.30, 0.30)	
	Abs. mean ± SD	0.62 ± 0.31	0.33 ± 0.22	0.62
	Abs. median (IQR)	0.60 (0.40, 0.90)	0.30 (0.15, 0.49)	

Abbreviations: Abs., absolute; CLD, central lung distance; HFD, heart shadow to field edge distance; IQR, interquartile range; SD, one standard deviation.

The mean ΔHFD was −0.62 cm with RPM, suggesting that on average heart appeared closer to (more towards or into) the field edge by 0.62 cm with RPM. The mean ΔHFD was small (−0.04 cm) with AlignRT, suggesting that there was no systematic displacement of the heart with AlignRT. The mean absolute displacement in HFD was larger with RPM (0.62 cm, median 0.60 cm) than with AlignRT (0.33 cm, median 0.30 cm), although this difference was not statistically significant. The correlation between HFD_DRR_ and HFD_PORT_ (Figure 4) were lower with RPM guidance (*R* ^2^ = 0.36, *r* = 0.60) than with AlignRT guidance (*R* ^2^ = 0.70, *r* = 0.84). As expected, there was no statistically significant difference from week to week in ΔCLD (*p* = 0.94) or ΔHFD (*p* = 0.30) since the repeated measurements of each displacement were related.

### 3.2 Displacement of chestwall and heart in VMAT patients

**FIGURE 5:**
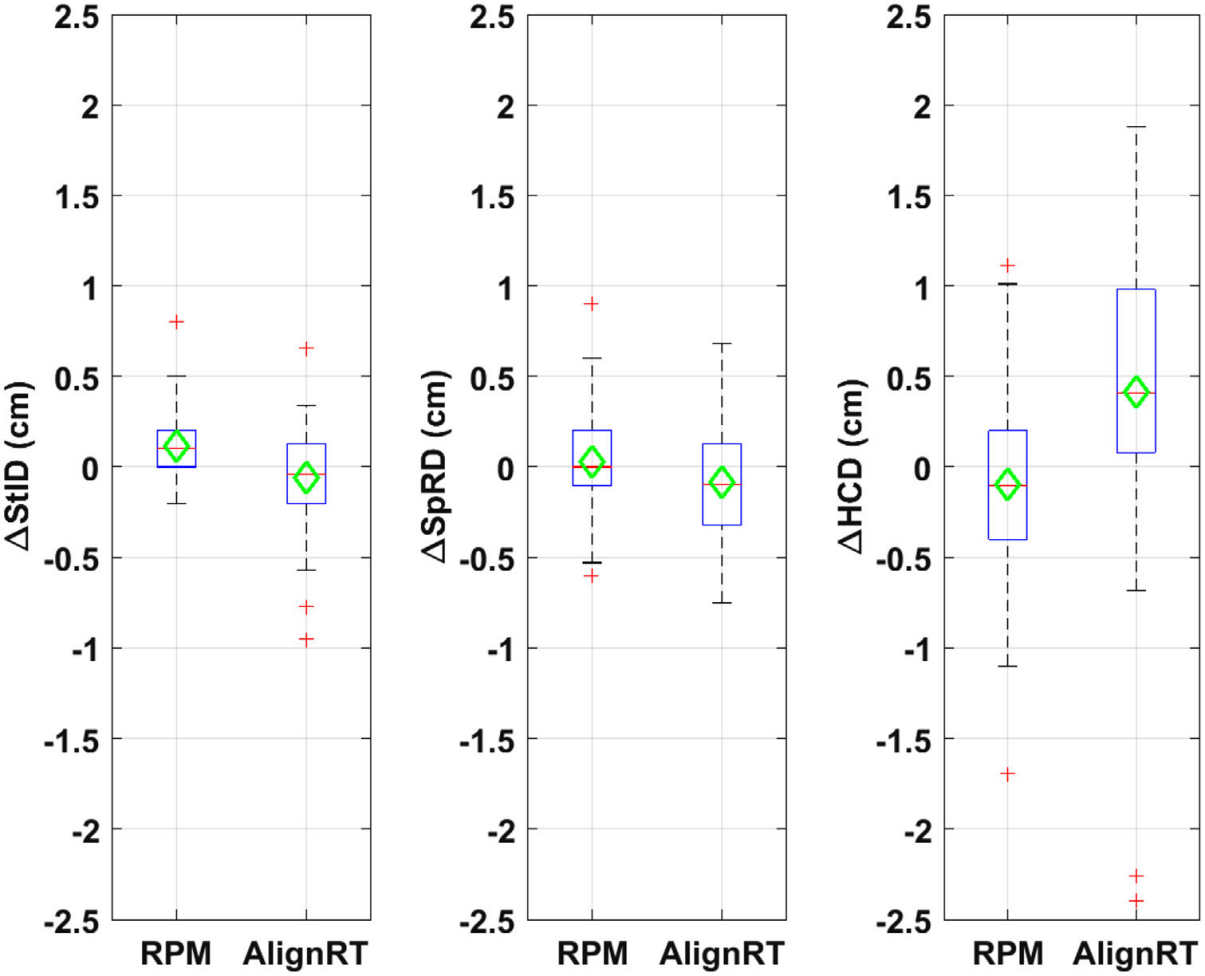
Box plots of ΔStID, ΔSpRD, and ΔHCD. The central line/marker indicates the median/mean, and the box indicates the 25th and 75th percentiles

**FIGURE 6:**
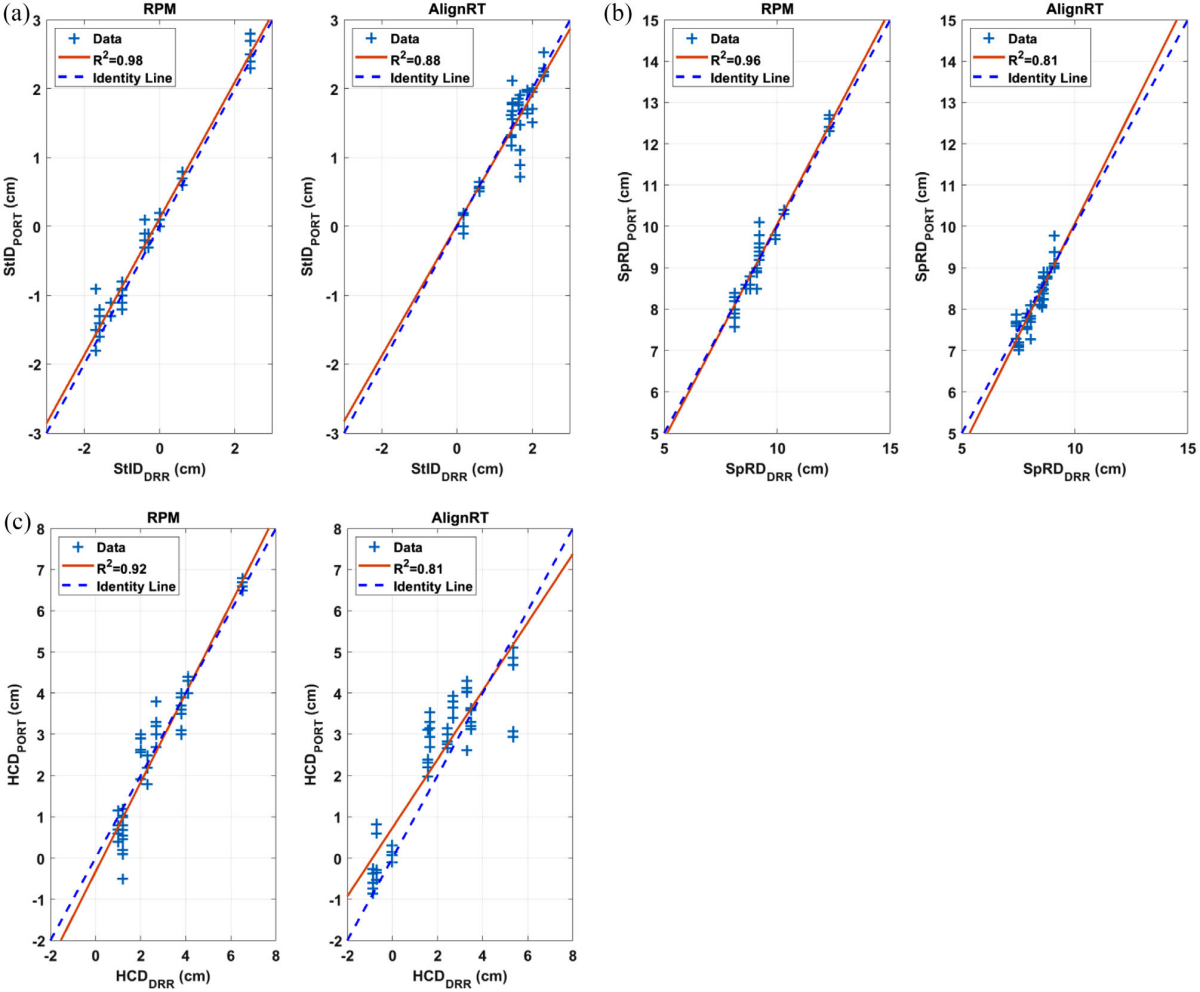
Correlation between (a) StID_DRR_ and StID_PORT_, (b) SpRD_DRR_ and SpRD_PORT_, and (c) HCD_DRR_ and HCD_PORT_ in volumetric modulated arc therapy (VMAT) patients

Table 2 and Figure 5 show the displacement in StID, SpRD, and HCD in VMAT patients. kV orthogonal images were acquired daily for 4–5 weeks. To reduce the data to be analyzed, only images on the first day and every five fractions afterward were included. This led to a set of 4–5 kV images for each patient, and a total of 48 and 50 sets of images for RPM and AlignRT patients, respectively. The results in Table 2 suggested that both the mean and mean absolute displacement in StID and SpRD were small and comparable between RPM and AlignRT. The correlations between StID_DRR_ and StID_PORT_ (Figure 6) and SpRD_DRR_ and SpRD_PORT_ were high with both RPM guidance (*R* ^2^ = 0.98, 0.96; *r* = 0.99, 0.98) and AlignRT guidance (*R* ^2^ = 0.88, 0.81; *r* = 0.94, 0.90).

**TABLE 2:** Displacement in volumetric modulated arc therapy (VMAT) patients

Displacement (cm)		RPM (*N* = 48)	AlignRT (*N* = 50)	*p*‐Value ANOVA
ΔStID	Mean ± SD	0.12 ± 0.17	−0.06 ± 0.28	0.08
	Median (IQR)	0.10 (0.00, 0.20)	−0.04 (−0.20, 0.13)	
	Abs. Mean	0.15 ± 0.15	0.21 ± 0.20	0.58
	Abs. Median (IQR)	0.10 (0.00, 0.20)	0.16 (0.08, 0.26)	
ΔSpRD	Mean ± SD	0.03 ± 0.26	−0.08 ± 0.28	0.17
	Median (IQR)	0.00 (‐0.10, 0.20)	−0.09 (−0.32, 0.13)	
	Abs. Mean ± SD	0.19 ± 0.18	0.24 ± 0.18	0.08
	Abs. Median (IQR)	0.14 (0.10, 0.20)	0.20 (0.10, 0.33)	
ΔHCD	Mean ± SD	−0.10 ± 0.54	0.41 ± 0.83	0.13
	Median (IQR)	−0.10 (−0.40, 0.20)	0.41 (0.08, 0.98)	
	Abs. Mean ± SD	0.41 ± 0.36	0.72 ± 0.58	0.12
	Abs. Median (IQR)	0.30 (0.10, 0.63)	0.62 (0.25, 1.33)	

Abbreviations: Abs., absolute; HCD, heart shadow to central axis distance (HCD); IQR, interquartile range; SD, one standard deviation; SpRD, spine to rib edge distance; StID, sternum to ISO distance.

The mean ΔHCD was −0.10 cm with RPM, suggesting that on average heart appeared closer to field CAX by 0.10 cm with RPM. Whereas the mean ΔHCD was 0.41 cm with AlignRT, suggesting that on average heart appeared 0.41 cm further away from CAX with AlignRT. The mean absolute displacement in HCD was smaller with RPM (0.41 cm) than with AlignRT (0.72 cm), although this difference was not statistically significant. The correlation between HCD_DRR_ and HCD_PORT_ (Figure 6) were high with both RPM guidance (*R* ^2^ = 0.92, *r* = 0.96) and AlignRT guidance (*R* ^2^ = 0.81, *r* = 0.90). As expected, there was no statistically significant difference from week to week in ΔStID, ΔSpRD or ΔHCD (*p* = 0.29, 0.91, 0.33, respectively) since the repeated measurements of each displacement were related.

### 3.3 Comparing displacement of chestwall and displacement of the heart

In both tangential patients and VMAT patients, the mean absolute displacement of chestwall or bony landmarks (∆CLD, ∆StID, ∆SpRD: 0.15–0.24 cm) were much smaller than those of heart (∆HFD, ∆HCD: 0.33–0.72 cm), with either RPM or SGRT. Also, the standard deviations (*k* = 1) in the displacement of chestwall (0.15–0.28 cm) were smaller than those of the heart (0.22–0.83 cm). The correlations between ∆CLD and ∆HFD in tangential patients were low: *r* = −0.33 with RPM and *r* = −0.21 with SGRT. The correlations between ∆StID and ∆HCD, and ∆SpRD and ∆HCD in VMAT patients were also low: *r* = −0.23, 0.11 respectively with RPM, and *r* = −0.04, 0.08, respectively, with SGRT.

## 4 DISCUSSION

In tangential field patients, RPM‐ and SGRT‐guided DIBH systems showed comparable and small mean absolute displacement difference of chestwall (∆CLD 0.23 vs. 0.19 cm). However, the displacement difference of the heart was larger (∆HFD 0.62 vs. 0.33 cm) and when compared to DRR, the heart appeared closer (−0.62 cm vs. −0.04 cm) to the field edge with RPM‐ than with surface‐guided DIBH. This difference in ∆HFD appeared large but was found not statistically significant because the ANOVA test for repeated measurements adequately treated the ∆HFDs measured weekly for the same patient as related measurements, and there were large variations within each small group of 10 patients. These observed better reproducibility of heart position with SGRT could be because (1) from FB to DIBH, the heart was pushed in 3D towards inferior, anterior, and medial directions. RPM monitored only the 1D motion of the small RPM reflective block (more specifically, the two reflective markers) along the AP direction. Whereas, the SGRT system monitored the 3D motion of a much larger body surface ROI. The RPM reflective block was distant (mean 8.1 cm, range 5.8–10.0 cm) from the most anterior “tip” of the heart shadow where we measured the HFD (Figure 1). Whereas the ROI in the SGRT system was large, the “tip” of the heart shadow and more than 50% of the heart lay beneath the body surface defined by the ROI. Therefore, the heart position might correlate better to the 3D motion of the large surface ROI than to the 1D motion of the small RPM reflective block.

In VMAT patients, RPM‐ and surface‐guided DIBH systems showed comparable and small mean absolute displacement difference of bony landmarks (∆StID 0.15 vs. 0.21 cm) and (∆SpRD 0.19 vs 0.24 cm). Interestingly, RPM‐guided DIBH showed a smaller (though not statistically different) difference in heart displacement (∆HCD 0.41 vs 0.72 cm) than the SGRT system.

RPM led to better reproducibility of heart position for VMAT patients than for tangential patients. We think a possible reason could be that the AP/LAT kV images in VMAT provided a 3D match to DRR whereas the MV portal images in tangential patients allowed only a 1D match (couch vertical) to DRR. That is, the 3D displacements were compensated for in both RPM (1D monitoring) and the SGRT system (3D monitoring) by using kV images in VMAT. On the other hand, the SGRT system led to worse reproducibility of heart position for VMAT patients than for tangential patients. A possible reason could be that bolus was used in all VMAT patients while only in one tangential patient. The presence of bolus could potentially reduce the correlation between the measured surface motion and internal motion (including heart motion) due to patient movement during bolus placement, lack of conformation of bolus to the body surface, and/or bolus moving and deforming differently than body surface as the patient progresses through the breathing cycle. On average heart appeared closer (−0.10 cm) to CAX with RPM whereas it appeared further (0.41 cm) away from CAX with SGRT.

The low correlations between chestwall displacement and heart displacement (3.3) indicated that heart position was not synchronized to chestwall position at DIBH. Therefore even when chestwall was well aligned, the heart might be misaligned. This was also observed in 10 tangential patients with RPM by McIntosh et al.,^37^ where rigid image registration was applied to align chestwall contours and heart contours respectively, between kV images and DRRs. They found a mean absolute displacement difference of 0.14 cm in the AP direction for chestwall, and mean absolute heart position deviations of 0.20, 0.16, and 0.25 cm in SI, Left/Right, and AP directions, respectively, with respect to chestwall.

Tang et al. reported their clinical experience using surface‐guided DIBH in 50 tangential patients^22^. They showed that the mean absolute ∆HFD was 0.20 cm with a correlation of *r* = 0.74. Our results (∆HFD = 0.33 cm, and *r* = 0.83) were similar to those results. In a small cohort of four tangential patients, Rong et al.^21^ acquired both RPM and SGRT breathing traces simultaneously. They showed that no correlation (*r* = −0.23 to −0.27) was found between the RPM displacement and the chestwall excursion, which was similar to CLD except that it was measured on real‐time MV Cine images acquired during treatment. In contrast, SGRT real‐time positioning offsets had a modest correlation with the chestwall excursion (*r* = 0.47–0.52). They concluded that the SGRT system provided a superior DIBH surrogate than RPM for tangential patients. A few studies compared external surface surrogate versus 1D respiratory signal in terms of their correlations with internal motion observed in 4DCT during free breathing, with mixed results. Fayad et al. studied 31 surface ROIs and 13 internal landmarks at various locations in 10 thoracic patients.^32^ They observed large differences in the motion correlation and hinted that with careful selection of ROI on the external surface, one could achieve a higher correlation than using a 1D respiratory signal like RPM. Fayad et al. showed in another study that using 3D surface information was superior to using both motion phase and amplitude extracted from a 1D respiratory signal in modeling patient‐specific respiratory motion.^33^ The difference was more substantial if only the phase or amplitude of the latter was used. One limitation of both Fayad et al. studies was that the external surface was obtained by segmenting the skin of all 4DCT volumes instead of by using a real‐time surface monitoring system. Kauweloa et al. compared the tracking accuracy of an SGRT system GateCT (single‐camera version of AlignRT) versus RPM system with a programmable respiratory motion platform simulating various motion amplitude, period, and irregularity.^30^ Their results showed that GateCT was not as accurate as RPM for amplitude ≤0.2 cm. Spadea et al. compared GateCT versus RPM system for 4DCT reconstruction with a sinusoidal motion phantom.^31^ They concluded that the two systems had similar performance, and there was no statistically significant difference in the reconstructed volumes of five moving spherical phantoms. One limitation in both phantom studies was that the surface for tracking was flat, that is, lack of curvatures or features, which was not a good representation of typical patient for SGRT systems. Whereas flat surface is not a limitation for RPM.

There are a few limitations in this study. First, the comparison between RPM and SGRT could not be made completely equivalent. They were compared in two different groups of patients at two sites within our institution. RPM monitored only the 1D motion of a small region while the SGRT system monitored the 3D motion of a much larger region. Also, the gating criteria were different between RPM (±0.25 cm in 1D) and SGRT (±0.3 cm translation and ±3.0° rotation in 6D). In another 10 patients whose RPM and SGRT signals were simultaneously acquired during treatment, we found that the two gating criteria had a Jaccard similarity index of 0.78, indicating moderate disagreement.^38^ Second, there was moderate to large uncertainty in measuring the heart distances. For some tangential patients, the boundary of the heart shadow in the MV portal image was blurred, resulting in uncertainty in measuring HFD (Figure 1d). The largest uncertainty was in measuring HCD (Figure 2e,f). HCD was sensitive to heart SI position. Although we measured HCD at the same SI vertebral position, the heart might be at different SI positions in the DRR than in the kV image due to variations in breath‐hold level and cardiac motion. Finally, although we observed some differences between RPM and SGRT, none of them were statistically significant, likely due to the small size of patient cohorts.

## 5 CONCLUSION

The displacements of chestwall (∆CLD, ∆StID and ∆SpRD) were small and were comparable with RPM‐ or SGRT‐guided DIBH. The position deviations of the heart were larger than the displacements of chestwall. Although none of the differences between RPM and SGRT was statistically significant, there was a trend that the position deviations of the heart were smaller and more favorable with SGRT‐guided DIBH than with RPM‐guided DIBH in tangential patients. These results supported more adoption of SRGT for breast radiotherapy while further study is needed.

## CONFLICT OF INTEREST

The authors declare that there is no conflict of interest that could be perceived as prejudicing the impartiality of the research reported.

## AUTHOR CONTRIBUTIONS

Dr. Lu had full access to all of the data in the study and takes responsibility of the integrity of the data and the accuracy of the data analysis. *Concept and design*: Lu, Tang, Li, Hong, Powell, Mechalakos, Zhang. *Acquisition, analysis, or interpretation of data*: Lu, Berry, Hong, Cerviño, Li, and Yorke. *Drafting of the manuscript*: Lu and Berry. *Critical revision of the manuscript for important intellectual content*: Lu, Hong, Cerviño, Yorke, Li, Berry, and Zhang. *Statistical analysis*: Lu, Berry, Hong. Administrative, technical, or material support: Berry, Tang, and Powell.
